# Potential of targeting signal-transducing adaptor protein-2 in cancer therapeutic applications

**DOI:** 10.37349/etat.2024.00216

**Published:** 2024-03-07

**Authors:** Taiga Maemoto, Yuto Sasaki, Fumiya Okuyama, Yuichi Kitai, Kenji Oritani, Tadashi Matsuda

**Affiliations:** Istituto Nazionale Tumori-IRCCS-Fondazione G. Pascale, Italy; ^1^Department of Immunology, Graduate School of Pharmaceutical Sciences, Hokkaido University, Sapporo 060-0812, Japan; ^2^Departmrnt of Hematology, International University of Health and Welfare, Narita 286-8686, Japan

**Keywords:** Signal-transducing adaptor protein, signal transduction, epidermal growth factor receptor, prostate cancer, lung cancer, peptides

## Abstract

Adaptor proteins play essential roles in various intracellular signaling pathways. Signal-transducing adaptor protein-2 (STAP-2) is an adaptor protein that possesses pleckstrin homology (PH) and Src homology 2 (SH2) domains, as well as a YXXQ signal transducer and activator of transcription 3 (STAT3)-binding motif in its C-terminal region. STAP-2 is also a substrate of breast tumor kinase (BRK). STAP-2/BRK expression is deregulated in breast cancers and enhances STAT3-dependent cell proliferation. In prostate cancer cells, STAP-2 interacts with and stabilizes epidermal growth factor receptor (EGFR) after stimulation, resulting in the upregulation of EGFR signaling, which contributes to cancer-cell proliferation and tumor progression. Therefore, inhibition of the interaction between STAP-2 and BRK/EGFR may be a possible therapeutic strategy for these cancers. For this purpose, peptides that interfere with STAP-2/BRK/EGFR binding may have great potential. Indeed, the identified peptide inhibitor successfully suppressed the STAP-2/EGFR protein interaction, EGFR stabilization, and cancer-cell growth. Furthermore, the peptide inhibitor suppressed tumor formation in human prostate- and lung-cancer cell lines in a murine xenograft model. This review focuses on the inhibitory peptide as a promising candidate for the treatment of prostate and lung cancers.

## Introduction

Most prostate cancer cases are responsive to androgen deprivation or anti-androgen therapy because tumor growth is highly dependent on androgens [1]. However, cancer-cell proliferation becomes resistant to androgen deprivation in some cases after long-term use of androgen-targeted drugs. This results in androgen-independent tumor growth progression, which can often be lethal. Epidermal growth factor receptor (EGFR) signaling, phosphatase and tensin homolog deletion from chromosome 10, and the activation of phosphatidylinositol 3-kinase (PI3K) and mitogen-activated protein kinase (MAPK) are important steps involved in androgen-independent tumor initiation/growth mechanisms [1]. Lung cancer is one of the leading causes of cancer-related deaths worldwide because only limited treatment options are available for its advanced-stage disease [2]. Most lung and non-small cell lung cancers are difficult to treat because of their poorly understood pathological mechanisms [3]. Recent progress in understanding cellular signal transduction pathways, including EGFR signaling (which controls cell survival), has identified genetic and regulatory abnormalities that suppress cell death, promote cell proliferation, and induce tumor formation [4].

Since EGFR signaling drives malignant transformation, EGFR-targeted drugs are used in clinical settings. Gefitinib is an EGFR tyrosine kinase inhibitor used to treat patients with cancer exhibiting *EGFR* mutations [5]. However, the efficacy of these drugs is limited [6], suggesting the involvement of unknown mechanisms. Therefore, further studies are necessary to explore the detailed mechanisms underlying EGFR-mediated cancer cell proliferation and tumor growth.

Signal-transducing adaptor protein-2 (STAP-2) is a macrophage colony-stimulating factor-1 (c-FMS)-binding protein containing a pleckstrin homology (PH) domain in its N-terminal region and an Src homology 2 (SH2) domain in its central region [7]. The SH2 domain of STAP-2 has 29% amino acid (aa) homology with human phospholipase C-2. STAP-2 contains a proline-rich region and a signal transducer and activator of transcription (STAT), STAT3-binding YXXQ motif, at its C-terminus. In line with STAP-2’s structure, we previously identified that STAP-2 interacts with various molecules and modifies their functions. The PH domain interacts with EGFR [8]. The SH2 domain interacts with IκB kinases (IKKs), myeloid differentiation primary response gene 88 (MyD88) [9], caspase-8 [10], or breakpoint cluster region-Abelson (BCR-ABL) [11]; both PH and SH2 domains interact with c-FMS [12], STAT5 [13], focal adhesion kinase (FAK)/proline-rich tyrosine kinase 2 (Pyk2) [14, 15], Casitas B-lineage lymphoma (CBL) [16], TNF receptor associated factor 3 (TRAF3) [17], and latent membrane protein 1 (LMP1) [17]; and the YXXQ motif interacts with STAT3 [7]. The fact that the STAP-2 PH and SH2 domains can bind to and modify several signaling molecules indicates that STAP-2 facilitates the initiation and progression of some cancer types. Therefore, inhibiting STAP-2 function may aid in developing anti-cancer drugs for various cancers. This review summarizes the functions of the previously developed STAP-2/EGFR-targeting inhibitory peptide *in vitro* and *in vivo*.

## Expression and function of STAP-2

**Figure 1:**
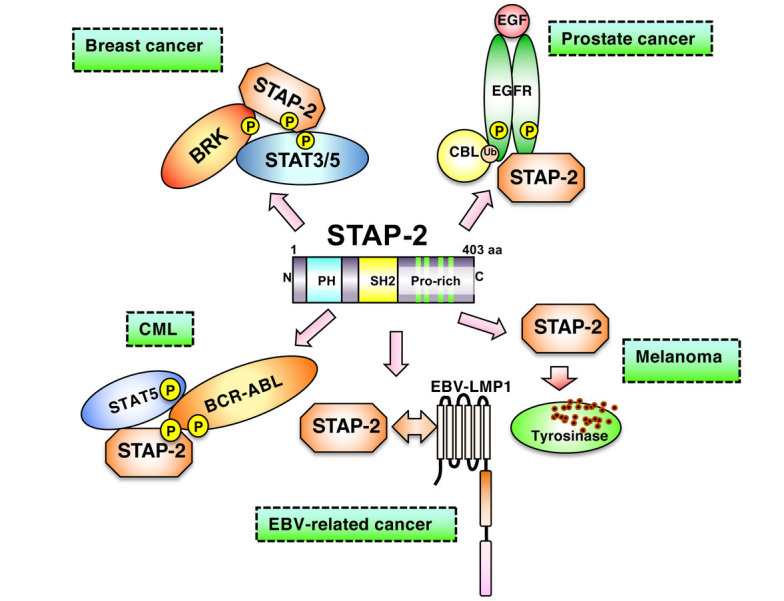
Involvement of STAP-2 in a variety of cancer cells. In breast cancer cells, STAP-2 acts as a scaffold protein to enhance BRK-mediated STAT3/5 activation. In prostate cancer cells, upon EFG binding to EGFR, STAP-2 helps EGFR stabilize its cell membrane without being degraded. In CML cells, STAP-2 mediates STAT5 activation through binding with BCR-ABL. In EBV-related cancer cells, STAP-2 interacts with EBV-LMP1 and promotes downstream signaling. STAP-2 prevents tyrosinase in lysosome from degradation in melanoma cells, modifying cancer phenotype. EGF: epidermal growth factor; P: phosphorylation; Ub: ubiquitination; EBV: Epstein Barr virus *Note.* Adapted from “Possible therapeutic applications of targeting STAP proteins in cancer,” by Matsuda T, Oritani K. Biol Pharm Bull. 2021;44:1810–18 (https://www.jstage.jst.go.jp/article/bpb/44/12/44_b21-00672/_html/-char/en). © 2021 The Pharmaceutical Society of Japan.

STAP-2 is expected to function in many cell types because it is widely expressed in various tissues and cells, such as lymphocytes, macrophages, and hepatocytes [7]. STAP-2 is also highly expressed in various cancer cells, including breast, prostate, lung cancer, melanoma, and leukemia. Among these cells, cancer cells express higher *STAP-2* mRNA expression than normal cells. In particular, prostate cancer cells express STAP-2 at high levels, similar to those in breast cancer cells [8]. In chronic myeloid leukemia cells, the interaction between STAP-2 and BCR-ABL is crucial in conferring growth advantages, resistance to imatinib (a BCR-ABL inhibitor), and tumor progression [11]. Thus, STAP-2 may affect various signaling and transcriptional molecules. In T cells, STAP-2 modulates STAT3- and STAT5-mediated expression of cytokine-related genes and enhances the activation of Fas cell surface death receptor (Fas)-induced apoptosis [18]. Additionally, STAP-2 upregulates Fc epsilon receptor I (FcRI)- and Toll-like receptor-mediated signals [18] in macrophages and dendritic cells. STAP-2 is also involved in chemokine- and integrin-mediated signaling [14, 19]. Therefore, STAP-2 may regulate both the immune and inflammatory responses. As shown in Figure 1, STAP-2 also binds to breast tumor kinase (BRK) and STAT3, resulting in the enhanced growth of T47D breast cancer cells [20]. In B16-F10 melanoma cells, STAP-2 positively regulates tyrosinase protein levels and modulates tumor invasion by regulating chemokine receptor expression [21]. In chronic myeloid leukemia cells, STAP-2 interacts with the fused BCR-ABL complex, resulting in the augmentation of its downstream signals [11].

## Effects of STAP-2 on EGFR-mediated signaling

After ligand binding, EGFR associates with growth factor receptor bound protein 2 (GRB2), followed by Rat sarcoma (RAS) activation. The RAS enhances extracellular signal-regulated kinase (ERK) activation, thereby inducing cancer-cell proliferation. Cell membrane EGFR expression is critical for RAS and ERK activation. STAP-2 upregulates EGFR-mediated signaling, enhancing STAT3 activity in DU145 prostate cancer cells [8]. After the ligation of EGFR to its ligands, the surface of EGFR is internalized and degraded in lysosomes. STAP-2 is associated with EGFR, and its binding suppresses EGFR internalization and degradation. EGFR stabilization by STAP-2 further enhances EGFR signaling and STAT3 upregulation. Activated EGFR is ubiquitinated by the E3 ubiquitin ligase CBL (c-CBL), resulting in its translocation from the plasma membrane to lysosomes. STAP-2 is a c-CBL substrate that aids in CBL-dependent protein degradation. EGF-induced Ub of EGFR increased in *STAP-2*-deficient cells but decreased in *STAP-2*-overexpressing prostate cancer DU145 cells. Additionally, increased EGFR trafficking to lysosomes was detected in *STAP-2*-deficient DU145 cells. BRK upregulates EGFR signaling by inhibiting CBL-mediated Ub of EGFR [22]. STAP-2 binds to BRK and CBL, thereby enhancing EGFR signaling via multiple mechanisms.

EGFR forms homodimers or heterodimers with human EGFR 2 (HER2), HER3, and HER4 upon ligand stimulation. Subsequently, dimerized EGFR activates downstream signaling molecules, including protein kinase B (AKT) and ERK. The CBL-promoted Ub of EGFR homodimers was faster than that of EGFR heterodimers. Hence, overactivation of EGFR signaling and enhanced tumor progression is commonly observed in *HER2*- or *HER3*-overexpressing cancer cells [23]. *STAP-2* knockdown negatively regulates the proliferation of DU145 and LNCaP prostate cancer cells [8]. *STAP-2* overexpression restores the surface expression of EGFR [8] but fails to interact with the EGFR K721A mutant lacking dimerization capacity. Therefore, the binding of STAP-2 to EGFR was restricted to EGFR dimer formation [8]. STAP-2 stabilizes normal EGFR but not mutant EGFR after EGF binding [8]. Moreover, *STAP-2* knockdown-induced suppression of tumor cell growth was observed in EGFR-activated, but not in EGFR-inactivated states [8]. Therefore, *STAP-2* inhibition can be developed as a therapeutic strategy for EGFR-related cancers.

## Peptides as protein-protein interaction inhibitors

As STAP-2/EGFR binding is an intracellular protein-protein interaction, small molecules or antibodies are physiochemically unsuitable for inhibiting this interaction [24–26]. Peptide drug research has progressed in recent years. Peptides are efficient tools for controlling protein-protein interactions, as they exhibit high stability in the blood and high affinity and permeability [27]. A recent study successfully developed a peptide targeting the interaction between programmed cell death protein 1 (PD-1) and programmed cell death protein ligand 1 (PD-L1), which activates the immune response and suppresses tumor progression [28]. Our previous study used peptides as interfering tools to inhibit protein-protein interactions and showed that the STAP-2 PH domain is a crucial site for EGFR binding [8]. We first synthesized several peptides containing the STAP-2 PH domain and octa-arginine (RRRRRRRRGG and R8) sequences in their N-terminal regions, to penetrate the cell membrane. We screened peptides based on the growth inhibition of the human prostate-cancer cell line DU145. The selected peptide was further optimized by shortening its sequence without affecting its inhibitory capacity [29]. A five-amino-acid sequence in the STAP-2 PH domain was identified as optimal. The optimized peptide and control peptide carrying the R8 sequence for permeability is referred to as “cell-penetrating STAP-2-derived synthetic peptide (2D5)” and “Ctrl,” respectively, in this review.

## Functional roles of the 2D5 peptide inhibitor *in vitro*

As STAP-2 is important for EGFR-mediated signaling, including the phosphorylation of EGFR, ERK, and STAT3 and proliferation of some human cancer-cell lines [8], we tested whether 2D5 suppresses these functions in a previous study. Using normal human, prostate/lung cancer (DU145/A375) and other cell lines, we showed that 2D5, but not Ctrl, inhibited EGFR-mediated signaling and cell proliferation [29]. As *STAP-2*-knockdown enhances the translocation of EGFR to lysosomes [8], we further investigated whether 2D5 treatment affects EGFR localization and degradation. Indeed, surface EGFR levels rapidly decreased, and EGFR translocation to lysosomes increased after EGF stimulation of 2D5-treated DU145 cells. However, 2D5 exerted inhibitory effects on murine cancer-cell lines (such as B16-F10, E0071, and EL-4) and human *EGFR*-negative cell lines [such as human T (Jurkat) and keratinocyte-like (HaCaT) cell lines]. Therefore, the inhibitory effects of 2D5 might be limited to human *EGFR*-positive cells.

The cytotoxic effects of 2D5 should be considered during drug development because drugs with high cytotoxicity exert various adverse effects [30]. We previously did not detect any cytotoxic effects of 2D5 on *EGFR*-negative cell lines, such as MDA-MB-453 and SW620 [29]. These findings suggest that 2D5 selectively inhibits *EGFR*-positive cancer cell growth by destabilizing surface EGFR proteins and downregulating EGFR signaling. Notably, 2D5 exhibited low cytotoxicity against *EGFR*-negative cells.

## Effects of 2D5 in a murine xenograft model

**Figure 2:**
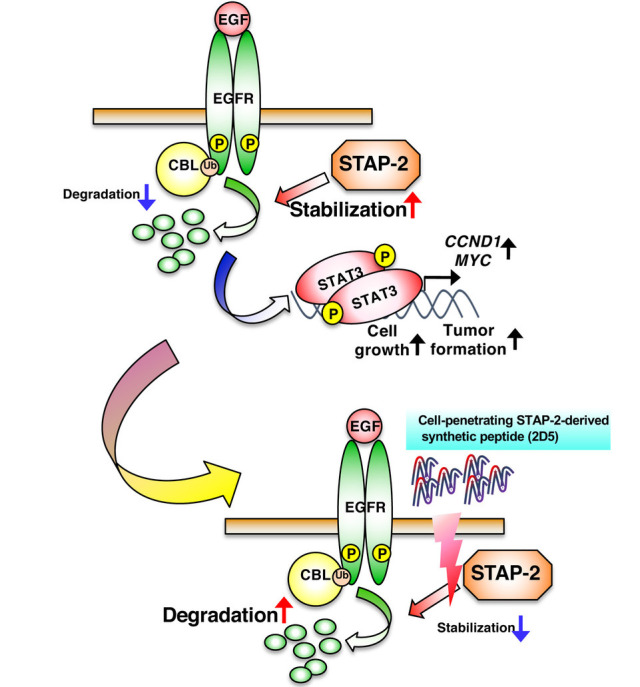
Suppression of EGFR-mediated signaling by the STAP-2-derived 2D5 peptide. 2D5 inhibits STAP-2/EGFR binding, followed by the suppression of EGFR stabilization and cancer-cell growth. CCND1: cyclin D1; MYC: myelocytomatosis *Note.* Adapted from “Possible therapeutic applications of targeting STAP proteins in cancer,” by Matsuda T, Oritani K. Biol Pharm Bull. 2021;44:1810–18 (https://www.jstage.jst.go.jp/article/bpb/44/12/44_b21-00672/_html/-char/en). © 2021 The Pharmaceutical Society of Japan.

Since 2D5 negatively regulates EGFR-mediated signaling and suppresses cell growth in human *EGFR*-positive cancer cell lines [29], we analyzed the therapeutic potential of 2D5 in cancer cells using a murine xenograft model. We observed that 2D5 significantly inhibited tumor formation and growth in DU145 and A549 cells, whereas Ctrl had no inhibitory effect on tumor progression. Furthermore, 2D5 exhibited no antitumor effects against *EGFR*-negative SW620 tumors in a murine xenograft model. In this xenograft model, 2D5 did not affect the body weight of mice, indicating its low toxicity *in vivo* [29]. Therefore, 2D5 inhibited tumor progression in prostate and lung cancers, suggesting its potential as a novel therapeutic agent for *EGFR*-positive cancers (Figure 2).

## Conclusions

Adaptor proteins are closely associated with various signaling pathways. STAP-2 regulates the immune and inflammatory responses and is involved in multiple malignancies [31–34]. Therefore, clarifying the underlying mechanisms through which intracellular signaling regulates cancer cell proliferation and tumor progression is important for identifying potential targets for clinical use. We previously showed that STAP-2 interacts with various intracellular signaling molecules, such as EGFR, MyD88, CD3ζ immunoreceptor tyrosine-based activation motif (ITAM), and Fas [9, 10, 12, 14–16, 19, 35–37]. STAP-2 binds to MyD88 in its SH2 domain and enhances cytokine production by upregulating IKK-mediated nuclear factor kappa B (NF-κB) activation. The 2D5 peptide did not affect lipopolysaccharide (LPS)-induced NF-κB activation in the human leukemia macrophage cell line, THP-1 [9]. STAP-2 also binds to CD3ζ ITAM in its proline-lich region and promotes the proliferation of human/murine T cells [35]. However, 2D5 does not affect the proliferation of Jurkat/EL-4 human/murine T cells [29]. Therefore, 2D5 selectively inhibits protein-protein interactions dependent on the STAP-2 PH domain, but not those dependent on the SH2 or proline-lich domain. Moreover, 2D5 blocked the proliferation of human cancer cells but not murine cancer cells. The 2D5 peptide sequence (excluding the cell-penetrating R8 sequence) exhibited 71% identity, whereas the EGFR cytosolic domain exhibited 96% identity between humans and mice. Furthermore, *STAP-2* knockdown suppresses the proliferation of B16-F10 cells, but the STAP-2/EGFR complex was not observed [29]. This suggested that murine B16-F10 cells undergo EGFR-independent proliferation. Alternatively, 2D5 might not inhibit the interaction between STAP-2 and EGFR in murine cells. Low cytotoxicity in non-cancerous cells and tissues is important for the development of anti-cancer drugs because it can cause severe side effects and even lead to death. In our previous study, 2D5 administration significantly inhibited cancer-cell proliferation and tumor progression in prostate and lung cancer cells *in vitro* and *in vivo.* However, non-cancer cells *in vitro* or the body weight of the *in vivo* xenograft model are not affected [29]. *STAP-2* knockout mice exhibit no abnormal phenotypes under steady-state conditions [35]. These results indicate that the 2D5 peptide can inhibit cancer-cell proliferation and tumor growth with high specificity and safety. EGFR-mediated signaling is overexpressed in various cancer cells but not normal cells [5, 38]. Cancer cells express proteins such as sortilin, tribbles homolog 3 protein (TRIB3), and ubiquitin specific peptidase 22 (USP22), which suppress EGFR degradation via EGFR stabilization, and their knockdown inhibits cancer cells [39–41]. These EGFR-stabilizing proteins are also promising targets for EGFR-overactivated cancer treatment. Therefore, future studies should investigate whether STAP-2 is associated with these proteins. In DU145 cells, half maximal inhibitory concentration (IC_50_) of 2D5 peptide was 19.4 μmol/L *in vitro* [29]. For application in clinical studies, 2D5 needs to be further optimized to improve its efficiency at low concentrations (submicromolar) *in vitro*. This can be achieved by increasing the stability and affinity of the peptides. Therefore, the 2D5 peptide could be used as a potential anti-cancer agent.

## Abbreviations

BCR-ABL: breakpoint cluster region-Abelson
BRK: breast tumor kinase
CBL: Casitas B-lineage lymphoma
EBV: Epstein Barr virus
EGF: epidermal growth factor
EGFR: epidermal growth factor receptor
ERK: extracellular signal-regulated kinase
HER2: human epidermal growth factor receptor 2
LMP1: latent membrane protein 1
PH: pleckstrin homology
RAS: Rat sarcoma
SH2: Src homology 2
STAP-2: signal-transducing adaptor protein-2
STAT3: signal transducer and activator of transcription 3
Ub: ubiquitination

## Declarations

### Copyright

© The Author(s) 2024.
